# Erfahrungen mit der orthopädischen Einlagenversorgung – Eine Querschnittsstudie

**DOI:** 10.1007/s00132-024-04476-9

**Published:** 2024-02-20

**Authors:** Tjorven Stamer, Minettchen Herchenröder, Malte W. Klee, Katja Götz, Jost Steinhäuser

**Affiliations:** Institut für Familienmedizin, Universitätsklinikum Schleswig-Holstein, Campus Lübeck, Ratzeburger Allee 160, 23538 Lübeck, Deutschland

**Keywords:** Querschnittsuntersuchung, Deutschland, Orthopädische Schuheinlagen, Orthesen, Fragebogen, Cross-sectional survey, Germany, Orthotic shoe inserts, Orthoses, Questionnaire

## Abstract

**Hintergrund:**

Orthopädische Einlagen (OE) werden zur Behandlung einer Vielzahl von Fußproblemen eingesetzt.

**Fragestellung:**

Ziel dieser Querschnittsstudie war es, die Wahrnehmungen zur Versorgung mit OE unter den Herstellern der OE, den Orthopädietechniker*innen (OT), zu untersuchen.

**Methodik:**

OT aus den Bundesländern Schleswig-Holstein und Niedersachsen in Deutschland wurden eingeladen, an einem Fragebogen teilzunehmen. Die Fragen umfassten, unter anderem, die Menge der Verordnungen zur Herstellung einer OE pro Monat sowie die verwendeten Materialien. Es wurden deskriptive Statistiken, Subgruppenanalysen sowie eine lineare Regressionsanalyse durchgeführt.

**Ergebnisse:**

Von den 312 verteilten Fragebögen wurden 159 vollständig ausgefüllt (Rücklaufquote 51 %). Die meisten der Befragten waren männlich (80 %). Das Durchschnittsalter lag bei 50 Jahren. Im Durchschnitt stellten die OT 290 OE pro Monat her, wobei Kunststoff das am häufigsten verwendete Material war (73 %). OT mit einer Berufserfahrung von weniger als 20 Jahren kommen bei der Herstellung von OE eher den ärztlichen Vorgaben nach als OT mit mehr als 20 Jahren Berufserfahrung. Letztere stützen ihre Entscheidungen bei der Herstellung sowie Ausgabe von OE eher auf eigene Erfahrungen.

**Schlussfolgerungen:**

Der Herstellungs- und Ausgabeprozess von OE gestaltet sich im Vergleich der OT unterschiedlich. Verschiedene berufliche Perspektiven der OT könnten hierbei eine Rolle spielen. Ebenso der Mangel eines standardisierten Vorgehens.

**Zusatzmaterial online:**

Zusätzliche Informationen sind in der Online-Version dieses Artikels (10.1007/s00132-024-04476-9) enthalten.

## Abstract (en)

**Background:**

Orthopaedic insoles (OI) are used to treat a variety of foot problems.

**Objectives:**

The aim of this cross-sectional study was to investigate the perceptions of the treatment with OI among the manufacturers of the OIs, the orthopaedic technicians (OT).

**Methods:**

OT from the federal states of Schleswig-Holstein and Lower Saxony in Germany were invited to take part in a survey. The questions included, among others, the number of prescriptions for the production of an OI per month and the materials used. Descriptive statistics, subgroup analyses and a linear regression analysis were performed.

**Results:**

Of the 312 questionnaires distributed, 159 were completed (response rate 51%). Most of the respondents were male (80%). The average age of the participants was 50. On average, OT produced 290 OI per month, with plastic being the most commonly used material (73%). OT with less than 20 years of professional experience are more likely to follow the doctor’s instructions when producing OI than OT with more than 20 years of professional experience. The latter are more likely to base their decisions regarding the manufacture and issuing of OI on their own experience.

**Conclusions:**

The production and issuing process of OI differs among OT. The different professional perspectives of the OT could play a role here, as does the lack of a standardized procedure. The factors listed in this study represent potential starting points for future research projects that could contribute to the development of evidence-based standards.

Orthopädische Einlagen (OE) werden für zahlreiche Fußbeschwerden empfohlen. 2022 erhielten in Deutschland ca. 4,5 Mio. Menschen (in etwa 5,4 % der Gesamtbevölkerung) ein Rezept für OE. OE gehören somit bundesweit zu den am häufigsten verordneten Medizinprodukten [1]. Ziel der Studie war es, die Erfahrungen, Herausforderungen sowie Optimierungsoptionen der Orthopädietechniker*innen (OT) in Bezug auf OE abzubilden.

Orthopädische Einlagen (OE) werden für zahlreiche Fußbeschwerden [2], darunter Platt- oder Senkfußschmerzen [3], Schmerzen im Bereich des Mittelfußes, der Ferse und Achillessehne, des Knies und der Hüfte [4, 5] sowie bei Rückenproblemen [6] empfohlen. OE gehören in Deutschland zu den am häufigsten verordneten Medizinprodukten [1, 7]. Für die gesetzlichen Krankenversicherungen bedeutete dies Kosten von über 528 Mio. € [1]. Trotz dieser Zahlen ist die Studienlage bezüglich der Wirksamkeit hierbei nicht eindeutig, wie verschiedene kürzlich erschienene systematische Reviews aufzeigen [8, 9]. Zu einer ähnlichen Schlussfolgerung kam ein Cochrane Review, wobei hier ausschließlich Kinder betrachtet wurden [10]. Mangelhaft erscheinen in diesem Zuge vor allem die verwendeten Studiendesigns [10].

Orthopädische Einlagen werden von Ärzt*innen verordnet [11], während sie von Orthopädietechniker*innen (OT) hergestellt und an die Patient*innen ausgegeben werden. Die Arbeit als OT ist ein traditioneller Handwerksberuf, der das Entwerfen, Entwickeln, Herstellen und Anpassen von verschiedenen orthopädischen und prothetischen Hilfsmitteln umfasst.

Für die Herstellung und Anpassung einer individuellen OE stehen verschiedene Möglichkeiten zur Verfügung: Z. B. ein zweidimensionaler (2-D) Fußabdruck [11], ein Gipsabdruck [12], ein dreidimensionaler (3-D) Fußabdruck [11, 13] oder ein Fußdruckmesssystem [11, 12].

Zu den Materialien, die üblicherweise für die Herstellung von OE verwendet werden, gehören Kork, Leder, Filz, Gummi und Kunststoffe [11]. Diese Materialien sind weich und biegsam und neigen unterschiedlich schnell dazu, zu verschleißen [12]. Weiterhin wird zwischen konventionell vorgefertigten [14], teilvorgefertigten [15] und maßgefertigten [16] OE unterschieden.

Ziel der vorliegenden Studie war es, die Erfahrungen von OT mit OE zu erheben.

Konkret sollten mit dieser Studie die folgenden zwei Teilfragen beantwortet werden:

1. Wie sind die Erfahrungen bezüglich des Herstellungsprozesses von OE?
2. Welche Probleme und Optimierungen sehen die Teilnehmenden im Zusammenhang mit OE?

## Methodik

### Design und Teilnehmende

Diese Querschnittsstudie wurde nach den STROBE-Richtlinien (Strengthening the Reporting of Observational Studies in Epidemiology) durchgeführt [17]. Sie fand in den norddeutschen Bundesländern Schleswig-Holstein und Niedersachsen statt.

Die OT erhielten eine Einladung zur Befragung in Form eines Briefes mit einem Link zum entsprechenden Online-Fragebogen auf Survey Monkey (SurveyMonkey Inc., San Mateo, Kalifornien, USA) [18]. Zusätzlich wurde eine Papierversion des Fragebogens an jeden/jede OT versandt. Die Adressen wurden von den Handwerkskammern übernommen [19, 20]. Insgesamt wurden im Zeitraum von Oktober bis Dezember 2021 312 Fragebögen verschickt. Die Rücksendung des anonymen Papierfragebogens oder das anonyme Ausfüllen des Online-Fragebogens auf der Webseite beinhaltete die Einverständniserklärung zur Teilnahme.

### Messung

Der Fragebogen wurde auf der Grundlage qualitativer Interviews, den Erfahrungen von zwei Physiotherapeutinnen (MH und K. Gwinner) [21], einem Allgemeinmediziner (JS) sowie den Empfehlungen einer australischen Studie [22] und einer systematischen Literaturrecherche entwickelt [23]. Anschließend wurde der Fragebogen pilotiert, aber nicht formell validiert. Die endgültige Umfrage umfasste 14 Fragen (siehe Anhang 1). Die Fragen bezogen sich, neben soziodemografischen Daten, auch auf die Verschreibung sowie die Herstellung von OE, das Alter der Gruppen, die am häufigsten OE erhalten, die Art der verordnenden Ärzt*innen, die am häufigsten verwendeten Materialien, die Überprüfung der hergestellten OE, mögliche weitere Therapiemaßnahmen und den Austausch mit anderen Berufsgruppen. Für die einzelnen Items wurden unterschiedliche Antwortmöglichkeiten genutzt. So mussten die Teilnehmenden zu den dargestellten Items frei gewählte Zahlen angeben, Mehrfachantworten wählen oder eine Zahl auf einer sechsstufigen Likert-Skala auswählen.

### Analyse der Daten

Die statistische Auswertung erfolgte mithilfe der Statistiksoftware SPSS 27.0 (IBM, Armonk, NY, USA, 2020). Zunächst wurden die Ergebnisse deskriptiv in Form von Mittelwerten, Standardabweichungen (SD) sowie Prozentsätzen erfasst. Subgruppenanalysen wurden mithilfe von Chi^2^-Tests für Geschlechtsunterschiede, Unterschiede in den Arbeitsgebieten (Stadt vs. Land) und Berufserfahrung durchgeführt. Die Berufserfahrung wurde in „20 Jahre und weniger“ und „mehr als 20 Jahre“ gruppiert. Dem wurde der Median zugrunde gelegt. Entsprechend konnten erfahrene mit sehr erfahrenen OT verglichen werden.

Im Anschluss an diese Analysen wurde eine schrittweise lineare Regressionsanalyse durchgeführt, um potenzielle Zusammenhänge zwischen der abhängigen Variable „spezifische Auswirkung der Verschreibung von OE“ und verschiedenen unabhängigen Variablen zu untersuchen, die signifikant mit dieser abhängigen Variable korrelierten. Es wurde auf Multikollinearität geprüft. Die Varianzinflationsfaktoren (VIF) sowie die Toleranzwerte des Regressionsmodells wurden angegeben. Die Werte für den VIF sollten nicht über 5,0 liegen, während die Toleranzwerte nicht unter 0,25 liegen sollten [24].

Für den Test der statistischen Signifikanz wurde ein Alpha-Niveau von *p* < 0,05 verwendet.

## Ergebnisse

Mit einer Rücklaufquote von 51 % nahmen 159 der 312 OT an der Umfrage teil. 80 % der Befragten waren männlich. Das Durchschnittsalter der Teilnehmer*innen lag bei 50 Jahren. Die meisten der Teilnehmer*innen kamen aus städtischen Gebieten (80 %).

Für Details siehe Tab. 1.

**Table 1:** 

Merkmale^a^		*Mittelwert (SD); min/max*
Alter (Jahre)		49,5 (11,4); 22/69
Arbeitserfahrung (Jahre)		27,7 (11,5); 2/50
**–**		*Anzahl (%)*
Geschlecht	Männlich	79 (80)
	Weiblich	20 (20)
Arbeitsbereich	Stadt	76 (79)
	Land	20 (21)
Bundesland	Schleswig-Holstein	36 (37)
	Niedersachsen	63 (63)

^a^*n* variiert aufgrund fehlender Daten

### Verschreibung von orthopädischen Einlagen und Altersgruppe

Im Durchschnitt stellten die OT 290 (SD = 470) neue OE pro Monat her, mit einem Höchstwert von 3000 und einem Mindestwert von 15. Auf Folgeverordnungen entfielen durchschnittlich 51 (SD = 22) Paare von OE.

OE wurden am häufigsten für die Gruppe der Erwachsenen zwischen 26 und 65 Jahren (59 %) ausgestellt, gefolgt von Senioren über 65 Jahren (2 %) und Jugendlichen zwischen 13 und 18 Jahren (0,6 %). Die übrigen teilnehmenden OT (38 %) gaben keine Altersgruppe an.

Mehr als die Hälfte der OT wurden von Orthopäd*innen (55 %) verschrieben, gefolgt von Allgemeinmediziner*innen (34 %), Neurolog*innen (7 %) und Kinderärzt*innen (6 %). Der Rest wurde von anderen Fachärzt*innen verschrieben (13 %).

### Herstellung

Die teilnehmenden OT berücksichtigten bei der Herstellung von OE die folgenden Aspekte: Sie inspizierten die Alltagsschuhe des/der Patient*in (98 %), sie beurteilten das Gangbild (88 %) und erhoben eine spezifische Anamnese (87 %). 81 % der OT tasteten die Schuhe ab und 70 % führten eine Inspektion durch, wenn sie OE anfertigten.

Bei der Diagnose Plattfuß war Kunststoff das am häufigsten verordnete Material (74 %), Kork das zweithäufigste (46 %), gefolgt von Leder (36 %) und Verbundwerkstoffen (17 %).

Welche Materialien bei der Herstellung von orthopädischen Verordnungen verwendet wurden, hing von den Schmerzen des/der Patient*in (91 %), den Erfahrungswerten der OT (82 %), der sportlichen Aktivität der Patient*innen (74 %), der beruflichen Belastung der Patient*innen (70 %) und der bisherigen Versorgung mit OE (70 %) ab.

### Einhalten der ärztlichen Vorgaben

Auf einer Skala von 1 („nie“) bis 6 („immer“) bewerteten die Teilnehmer*innen mit einer 2 (SD = 0,9), wie stark sie sich im Durchschnitt an die Vorgaben der Ärzt*innen hielten.

Faktoren, die einen Einfluss auf diese Befolgung hatten, waren die technischen Erfahrungswerte der OT (70 %), die wahrgenommene Kompetenz der verordnenden Ärzt*innen (53 %), die Bereitschaft der Patient*innen zur Zuzahlung (43 %) und die Kooperationsfähigkeit des verordnenden Arztes/der verordnenden Ärztin (37 %).

### Ausgabe der orthopädischen Einlagen

Eine detaillierte Übersicht, worauf bei der Herausgabe von OE geachtet wurde, ist in Tab. 2 dargestellt.

**Table 2:** 

Schritte bei der Ausgabe	*N* (%)
Kontrolle der Verarbeitung	91 (90)
Anlegen der Schuheinlagen am unbelasteten Fuß	74 (73)
Zufriedenheit des/der Patient*in	74 (70)
Ganganalyse	71 (50)
Anlegen der Schuheinlagen am belasteten Fuß	68 (68)
Abtasten des Einlagenmaterials	25 (25)

Auf einer Skala von 1 („nie“) bis 6 („immer“) wurde mit 2,8 (SD = 1,7) angegeben, wie oft im Durchschnitt Patient*innen einen Kontrolltermin zur erneuten Kontrolle der OE erhalten haben.

### Therapieerfolg und therapeutische Messung

Die spezifische Wirkung von OE wurde von den Teilnehmer*innen mit 78 % (SD = 20) eingeschätzt, wobei die maximale Schätzung bei 100 % und die minimale Schätzung bei 5 % lag.

### Zusammenarbeit

Bei einer notwendigen Verordnungskorrektur (76 %), bei komplexen Fällen, die ein Brainstorming erfordern (61 %) und bei einer notwendigen OE-Korrektur (42 %) wurde der Austausch mit anderen Berufsgruppen (Hausarzt/Hausärztin, Orthopäd*in oder Physiotherapeut*in) gesucht.

### Optimierungspotenzial

Die Teilnehmer*innen sprachen sich in 15 % für eine optimierte interdisziplinäre Zusammenarbeit aus. Eine vollständige Übernahme von OE durch die Krankenkassen (14 %) und ein einheitlicher Erstattungs- und Indikationskatalog für alle Krankenkassen (12 %) waren weitere Aspekte für Optimierungen (Tab. 3).

**Table 3:** 

Untersuchungsmethoden	Anzahl (%)
Optimierte interdisziplinäre Zusammenarbeit	49 (51)
Volle Kostenübernahme durch die Krankenkassen	47 (49)
Einheitlicher Erstattungs- und Indikationskatalog der Krankenkassen	39 (40)
Mehr Studien zur Evidenz von Schuheinlagen	36 (37)
Industriefreie interdisziplinäre Ausbildung für die Einlagenversorgung	33 (34)
Etablierung der Schuheinlagenversorgung in der Ausbildung	32 (33)
Standardisierte Richtlinien für Einlagenverordnungen	27 (28)
Vorhandensein eines definierten Behandlungspfades im Zusammenhang mit Einlagenverordnungen	26 (27)
Optimierung der Therapietreue der Patient*innen	18 (19)
Keine Zuzahlung durch Patient*innen	15 (16)
Standardisierte Einlagenherstellung durch Orthopädietechniker*innen	4 (4)

### Subgruppenanalysen

Männliche OT (65 % der Männer) achteten mehr auf die Ökonomie bei der Herstellung von OE (*p* = 0,012) als weibliche OT (35 % der Frauen). Außerdem führte ein höherer Prozentsatz der männlichen OT (85 % der Männer) bei der Herstellung der OE eine Fußpalpation durch als bei den weiblichen OT (60 % der Frauen) (*p* = 0,014).

Wenn die Patient*innen keine Abneigung gegen eine Zuzahlung bei der Herstellung von OE zeigten, hielten sich die OT in ländlichen Gebieten (65 % der ländlichen Stichprobe) eher (*p* = 0,031) an die Anweisungen der Ärzt*innen als die in der Stadt arbeitenden OT (38 % der städtischen Stichprobe). In ländlichen Gebieten (90 % der ländlichen Stichprobe) führten OT auch häufiger eine Ganganalyse durch (*p* = 0,034) als OT in städtischen Gebieten (66 % der städtischen Stichprobe).

OT mit einer Berufserfahrung von 20 Jahren oder weniger hielten sich häufiger an ärztliche Vorgaben als OT mit sehr langer Berufserfahrung (*p* = 0,032). Hierbei relevant ist der Faktor der Kooperationsfähigkeit der Ärzt*innen.

Weiterhin wurde festgestellt, dass OT, die 20 Jahre oder weniger gearbeitet hatten, eher die vorhergegangene OE-Versorgung der Patient*innen berücksichtigten (*p* = 0,032) als diejenigen, die mehr als 20 Jahre gearbeitet hatten.

### Schätzung der spezifischen Wirkung auf die Verschreibung von Schuheinlagen

Es wurde ein vierstufiges Regressionsmodell durchgeführt, das 29,1 % (R^2^ ~ 0,291) der Varianz der Zielvariable „Schätzung der spezifischen Wirkung der Verschreibung von OE“ erklärt. Koeffizienten, die im Laufe der Analyse in das Modell aufgenommen wurden, waren „Kontrolltermin zur Nachkontrolle der Einlagen“ sowie „Berücksichtigung der Ergebnisse der durchgeführten Messung“.

Eine detaillierte Beschreibung der schrittweisen linearen Regression findet sich in Tab. 4.

**Table 4:** 

	Schritt 1	Schritt 2	Schritt 3	Schritt 4	VIF-Wert	Toleranzwert
Alter	0,372	0,373	0,346	0,309	1,00	1,00
Komorbidität der Patient*innen	–	0,298	0,279	0,194	1,00	1,00
Kontrolltermin zur Nachkontrolle der Einlagen	–	–	−0,246	−0,259	0,98	1,02
Berücksichtigung der Ergebnisse der durchgeführten Messung	–	–	–	0,209	0,80	1,24
R^2^	0,129	0,210	0,263	0,291	–	–

Es wurden nur Koeffizienten mit einer statistischen Signifikanz auf dem Niveau α < 0,05 angegeben

## Diskussion

Es handelt sich um die erste Querschnittsstudie, an der Orthopädietechniker*innen aus zwei Bundesländern in Deutschland beteiligt waren. Es gibt mehrere Fachärzt*innen, die an der Verschreibung von OE beteiligt sind, aber nur eine Berufsgruppe, die sie herstellt und ausgibt.

Unsere Daten zeigen, dass etwa der gleiche Prozentsatz der Befragten aus städtischen oder ländlichen OT stammt. Die soziodemografischen Ergebnisse hinsichtlich des Geschlechts waren in etwa mit den offiziellen Daten vergleichbar [25].

Während die papierbasierte Befragung eine Rücklaufquote von 26 % aufwies, lag die Rücklaufquote bei der Online-Version bei 74 %. Diese hohe Beteiligung an der Online-Umfrage lässt sich möglicherweise dadurch erklären, dass eine große Anzahl eher jüngerer und entsprechend technikaffiner Teilnehmer*innen an der Studie teilnahm und sich im Zuge dessen für das digitale Vorgehen entschied.

In Bezug auf die oben genannten Forschungsfragen zeigen unsere Ergebnisse, dass die OT eine hohe Anzahl von OE in einem einzigen Monat herstellen und dass Kunststoff das am häufigsten verwendete Material ist. Dies stimmt mit den Ergebnissen anderer vergleichbarer Studien überein [26, 27].

Weiterhin bilden unsere Ergebnisse ab, dass OT, die weniger als 20 Jahre Berufserfahrung angeben, eher der Verordnung der Ärzt*innen nachkommen, als diejenigen, die mehr als 20 Jahre Erfahrung haben. Diese Diskrepanz könnte darauf zurückzuführen sein, dass unterschiedliche berufliche Perspektiven bei der Herstellung von OE eine Rolle spielen. Ein zweiter Grund könnte darin liegen, dass keine Standards zum konkreten Vorgehen bestehen. Inwieweit Defizite in der Aus- und Weiterbildung von Ärzt*innen dieses Ergebnis mitbeeinflussen, sollte Gegenstand zukünftiger Forschung sein. Bisher können OT bei den Entscheidungen über die Herstellung von OE vor allem auf ihre eigenen Erfahrungen zurückgreifen [28]. Hier wären Studien wünschenswert, um zukünftig evidenzbasiert vorgehen zu können.

Passend zu Vorarbeiten [29] verdeutlicht unsere Studie, dass kein geregelter diagnostischer Weg hin zur OE existiert [12, 22] und dass nach wie vor ein Mangel an hochwertigen Studien zur Wirksamkeit und zum Therapieerfolg von OE besteht.

Entsprechend ist es plausibel, dass die interdisziplinäre Zusammenarbeit bei steigender Komplexität eines Falles zunehmend an Bedeutung gewinnt [30].

Vor dem Hintergrund der häufigen Angaben, dass sich nicht an eine ärztliche Vorgabe gehalten wurde, sollten ergänzende qualitative Studien durchgeführt werden, um zu thematisieren, welche Fälle von den Teilnehmenden bereits als ein Fall von sich nicht an eine ärztliche Vorgabe halten interpretiert werden.

### Stärken und Limitationen

Unsere Umfrage bei den OT war die erste ihrer Art in Deutschland und umfasste eine Stichprobe aus Teilnehmer*innen mit einem hohen Maß an Berufserfahrung, welche Subgruppenvergleiche bezüglich sehr geringer bzw. geringer Berufserfahrung erschwert. Über die Einteilung der Berufserfahrung mittels der Berechnung des Medians wurde eine erste Annäherung an diese Thematik getroffen. Bisher sind keine vergleichbaren Daten veröffentlicht worden.

Einige Fragen hatten trotz Pilotierung einen hohen Grad an fehlenden Werten, d. h. die Teilnehmer*innen haben sich entschieden, eine Frage mindestens zum Teil nicht zu beantworten. Beispielsweise ist die Frage nach der spezifischen oder unspezifischen Wirkung der OE-Verordnung im Fragebogen möglicherweise nicht eindeutig und verständlich genug definiert worden. Weiterhin handelte es sich um subjektive Angaben der Befragten, was sich unter anderem daran zeigte, dass eine Person angab, mehr als 3000 Einlagenverordnungen in einem durchschnittlichen Monat zu erhalten.

Um die Sichtweise der Gesamtheit der OT aufzuzeigen, sind daher weitere Studien notwendig, die mehr Bundesländer in Deutschland mit einem zuvor formal validierten Fragebogen einbeziehen sollen. Zudem wurde keine Power-Berechnung durchgeführt. Zuletzt sei anzumerken, dass es sich bei dieser Studie um eine Querschnittsstudie handelt, sodass mit der Ableitung von Kausalzusammenhängen aus diesen Ergebnissen vorsichtig umgegangen werden muss.

## Ausblick

Die Ergebnisse dieser Studie weisen auf die Notwendigkeit hin, mehr Evidenz für die Verschreibung und Herstellung von OE zu schaffen. Dadurch könnte die Behandlung mit OE für die meisten der häufigen Fußprobleme standardisiert und so optimiert werden.

Darüber hinaus kann dadurch die Möglichkeit eines einheitlichen Erstattungs- und Indikationskatalogs für die Krankenkassen geschaffen werden, wie er von den Teilnehmer*innen gewünscht wurde.

## Fazit für die Praxis

- Fußorthesen gehören, trotz unklarer Evidenz, zu den am häufigsten verordneten Medizinprodukten in Deutschland.
- Die Wahrnehmungen und Herausforderungen der Orthopädietechniker*innen im Rahmen des Herstellungs- und Ausgabeprozesses werden in der vorliegenden Arbeit erstmalig dargestellt.
- Eine umfassendere evidenzbasierte Grundlage für die Verschreibung, Herstellung und Ausgabe von Fußorthesen könnte die Behandlung der häufigsten Fußprobleme optimieren.
- Eine standardisierte, evidenzbasierte Vorgehensweise im Rahmen der Behandlung mit Fußorthesen wird empfohlen.
- Weitergehend würde auf Basis der potenziellen evidenzbasierten Standardisierung die Möglichkeit eines einheitlichen Erstattungs- und Indikationskatalogs für die Krankenkassen geschaffen werden.

### Supplementary Information





## Abkürzungen

OE: Orthopädische Einlagen
OT: Orthopädietechniker*innen
SD: Standardabweichung
VIF: Varianzinflationsfaktor
