# Supplementary material for: Experience with orthopaedic insoles—a cross-sectional study
Source: Orthopadie (Heidelb). 2024 Feb 20;53(4):291–6. [Article in German] doi: 10.1007/s00132-024-04476-9 (PMC10978688; doi:10.1007/s00132-024-04476-9)
Supplement: Supplementary file 1 [file 132_2024_4476_MOESM1_ESM.pdf]

UNIVERSITÄT ZU LÜBECK

Orthopädische Einlagen-eine Befragung unter Orthopädietechniker*innen

Sehr geehrte Damen und Herren,

das Institut für Allgemeinmedizin des Universitätsklinikums Schleswig-Holstein am Campus Lübeck lädt Sie ein, an einer Befragung zu **orthopädischen Einlagen** teilzunehmen.

Durch Ihre Angaben helfen Sie uns, einen Überblick über die Verordnung und Herstellung orthopädischer Einlagen zu bekommen.

Die Teilnahme an der Befragung wird etwa 10 Minuten dauern. Mit dem Zurücksenden des Fragebogens willigen Sie ein, dass Ihre Angaben durch das Institut für Allgemeinmedizin ausgewertet und publiziert werden dürfen. Das Ausfüllen des Fragebogens erfolgt anonym. Dritte erhalten keine Einsicht in die Originalunterlagen.

Vielen Dank für Ihre Unterstützung!

1a. Wie häufig erhalten Sie eine Verordnung zur Herstellung von orthopädischen Einlagen in einem durchschnittlichen Monat?

Angabe: ____

1b. Wie viel Prozent der Verordnungen sind davon Folgeverordnungen?

Angabe: ☐ ☐ ☐ %

2. Für welche Altersgruppe erstellen Sie am häufigsten Einlagen? (**eine** Nennung möglich)

- ❑ Kinder (bis 12 Jahre)
- ❑ Jugendliche (13-18 Jahre)
- ❑ junge Erwachsene (19-25 Jahre)
- ❑ Erwachsene (26-65 Jahre)
- ❑ Senioren (über 65 Jahre)

3. Wie viel Prozent der Verordnungen erfolgen durch …

Hausärzt*innen: ☐ ☐ ☐ %

Orthopäd*innen: ☐ ☐ ☐ %

Pädiater*innen: ☐ ☐ ☐ %

Neurolog*innen: ☐ ☐ ☐ %

Sonstige, und zwar: __________________

4. Welche der folgenden Aspekte berücksichtigen Sie bei der Herstellung orthopädischer Einlagen? (Mehrfachnennungen möglich)

❑	Ich erhebe die Anamnese.
❑	Ich führe die Inspektion durch.
❑	Ich führe die Palpation durch.

❑	Ich beachte die Alltagsschuhe.
❑	Ich führe Beweglichkeitstests durch.
❑	Ich schaue mir den Gang der Patient*innen an.
❑	Ich führe eine Fußdruckmessung (z.B. Pedobarographie) durch.
❑	Ich führe eine Messung der Bodenreaktionskräfte durch.
❑	Ich führe einen Trittschaumabdruck durch.
❑	Ich erstelle einen Fußabdruck mit Papier und Tinte.
❑	Ich erstelle einen Gipsabdruck.
❑	Ich beziehe die Wirtschaftlichkeit mit ein.
❑	Ich halte Rücksprache mit den verordneten Ärzt*innen.
❑	Sonstiges, und zwar:

5. **Welches Material für eine Einlage bei der Indikation „Plattfuß“ wird am häufigsten verordnet?** (Mehrfachnennungen möglich)

❑ Kork

❑ Leder

❑ thermoplastische Kunststoffe

❑ Faserverbundwerkstoffe

❑ Anderes Material, und zwar: ____

6. **Wie häufig halten Sie sich an die ärztlichen Vorgaben für die Herstellung von Einlagen?**

❑	❑	❑	❑	❑	❑
immer					nie

7. **Von welchen Faktoren machen Sie es abhängig, inwieweit Sie sich an die ärztlichen Vorgaben halten?** (Mehrfachnennungen möglich)

❑	Kompetenz der verordneten Ärzt*innen	❑	Kooperationsfähigkeit der verordneten Ärzt*innen	❑	Meine technischen Erfahrungswerte

❑	Verfügbarkeit der Materialien	❑	Krankenkasse	❑	Wirtschaftlichkeit
❑	Zuzahlungsbereitschaft der Patient*innen	❑	Anderes Kriterium, und zwar:		

8. **Welche Kriterien berücksichtigen Sie neben der Indikation für Ihre Entscheidung, welche Materialien und Besonderheiten (z. B. Pelotte oder Aussparungen) Sie für die Herstellung der Einlage für Patient*innen verwenden?** (Mehrfachnennungen möglich)

❑	Alter der Patient*innen	❑	Berufliche Belastung der Patient*innen	❑	Bisherige Einlagenversorgung der Patient*innen
❑	Meine Erfahrungswerte	❑	Ergebnisse der durchgeführten Messungen	❑	Erwartungen der Patient*innen
❑	Gangbild der Patient*innen	❑	Komorbidität der Patient*innen	❑	Schmerzen der Patient*innen
❑	Sportliche Betätigung der Patient*innen	❑	Verordnung	❑	Wirtschaftlichkeit
❑	Anderes Kriterium, und zwar:				

9. **Welche Schritte führen Sie ggf. bei der Ausgabe der Einlage an die Patient*innen durch?** (Mehrfachnennungen möglich)

❑	Kontrolle der Ein- und Ausarbeitungen (z.B. Pelotte)	❑	Palpation des Einlagenmaterials	❑	Anlegen der Einlage an den unbelasteten Fuß
❑	Anlegen der Einlage an den belasteten Fuß	❑	Ganganalyse mit Einlage	❑	Erhebung der Patient*innen-zufriedenheit
❑	Ich führe keine weiteren Schritte durch.	❑	Anderes Vorgehen, und zwar:		

10. **Wie häufig vereinbaren Sie mit den Patient*innen einen Kontrolltermin zur erneuten Überprüfung der Einlage?**

❑	❑	❑	❑	❑	❑
immer					nie

11. Was schätzen Sie: Wie viel Prozent der Effektstärke einer Einlagenverordnung liegt an <u>spezifischen Effekten</u> der Einlage? (Im Gegensatz zu unspezifischen Effekten („Placeboeffekte“))

Angabe: ☐ ☐ ☐ %

12. Welche ergänzenden Therapiemaßnahmen empfehlen Sie zusätzlich zu einer Einlagenverordnung?

❑ Ich empfehle keine weiteren Therapiemaßnahmen.

❑ Ich empfehle weitere Therapiemaßnahmen.

Bitte beschreiben Sie die „weiteren Therapiemaßnahmen“ genauer:

____

13. In welchen Fällen suchen Sie ggf. den Austausch mit anderen Berufsgruppen im Zusammenhang mit Einlagenverordnungen? (<u>Mehrfachnennungen möglich</u>)

❑	Notwendige Verordnungskorrektur	❑	Notwendige Einlagenkorrektur	❑	Brainstorming bei komplexeren Fällen
❑	Ich suche keinen Austausch.	❑	Anderes Thema und zwar:		

14. Welche Optimierungen bezüglich Einlagenverordnungen würden Sie am meisten begrüßen? (<u>bitte wählen Sie **maximal** drei Aspekte aus</u>)

❑	Vorhandensein eines definierten Behandlungspfades im Zusammenhang mit Einlagenverordnungen
❑	Mehr Studien zur Evidenz von Einlagen
❑	Standardisierte Leitlinien für Einlagenverordnungen
❑	Optimierung der Therapieadhärenz[1] der Patient*innen
❑	Keine Zuzahlung der Patient*innen
❑	Standardisierte Einlagenfertigung der Orthopädietechniker*innen
❑	Etablierung der Einlagenversorgung in die Ausbildung

❑	Industriefreie interdisziplinäre Fortbildungen für Einlagenversorgungen
❑	Optimierte interdisziplinäre Zusammenarbeit
❑	Einheitlicher Vergütungs- und Indikationskatalog der Krankenkassen
❑	Übernahme aller Kosten durch die Krankenkassen
❑	Sonstiges, und zwar:

[1] Einhaltung der gemeinsam von Patient*innen und Ärzt*innen verabredeten Therapieziele

Angaben zur Person

I. Ich bin… ❑ männlich ❑ weiblich ❑ divers

II. In welchem Jahr wurden Sie geboren? ☐☐☐☐

III. Wo praktizieren Sie? ❑ in der Stadt ❑ auf dem Land

IV. In welchem Bundesland sind Sie tätig? ❑ Schleswig-Holstein ❑ Niedersachsen

V. Seit wie vielen Jahren praktizieren Sie insgesamt? ☐☐

Welcher Aspekt zum Thema Einlagenverordnung ist Ihnen noch wichtig, den wir in dieser Befragung bisher nicht angesprochen haben?

(Bitte deutlich schreiben!)

Vielen Dank für Ihre Teilnahme!
